# Supplementary material for: Peripheral blood immune cell parameters in patients with high-grade squamous intraepithelial lesion (HSIL) and cervical cancer and their clinical value: a retrospective study
Source: PeerJ. 2024 Jun 3;12:e17499. doi: 10.7717/peerj.17499 (PMC11155673; doi:10.7717/peerj.17499)
Supplement: Supplemental Information 2 [file peerj-12-17499-s002.docx]

STROBE Statement—checklist of items that should be included in reports of observational studies

|  | Item No. | Recommendation | Page  No. | Relevant text from manuscript |
| --- | --- | --- | --- | --- |
| **Title and abstract** | 1 | (*a*) Indicate the study’s design with a commonly used term in the title or the abstract | 1 | To investigate the levels of peripheral blood lymphocyte parameters in patients with high-grade squamous intraepithelial lesion ( HSIL ) and cervical cancer, and to analyze the relationship between peripheral blood lymphocyte parameters and clinicopathological features in patients with cervical cancer. |
|  |  | (*b*) Provide in the abstract an informative and balanced summary of what was done and what was found | 1-2 | In this study, a retrospective case-control study was conducted. It was found that the expression levels of NLR and PLR in peripheral blood of patients with high-grade squamous intraepithelial lesion ( HSIL ) and cervical cancer were higher, and the levels of NLR and PLR in peripheral blood of patients with cervical cancer were closely related to clinical stage and infiltration of muscle wall. |
| Introduction | | | |  |
| Background/rationale | 2 | Explain the scientific background and rationale for the investigation being reported | 2-3 | Neutrophil lymphocyte ratio ( NLR ) is a reliable index to evaluate individual immune inflammatory state. Platelet lymphocyte ratio ( PLR ) is an important biomarker to reflect the inflammatory response and immune function in vivo, which is closely related to the occurrence and development of malignant tumors. At present, there are few reports on the levels of NLR and PLR in peripheral blood of HSIL and cervical cancer. |
| Objectives | 3 | State specific objectives, including any prespecified hypotheses | 2-3 | In this study, the levels of NLR and PLR in peripheral blood of patients with high-grade squamous intraepithelial lesion ( HSIL ) and cervical cancer were detected, and the relationship between them and clinicopathological features of cervical cancer was evaluated, so as to provide basis for clinical diagnosis and treatment of cervical cancer. |
| Methods | | | |  |
| Study design | 4 | Present key elements of study design early in the paper | 3 | This study used a retrospective case-control study. Thirty-nine patients with HSIL and 42 patients with cervical cancer who were treated in our hospital from July 2020 to September 2023 were selected, and 31 women who underwent physical examination in our hospital at the same time were selected as the healthy control group. |
| Setting | 5 | Describe the setting, locations, and relevant dates, including periods of recruitment, exposure, follow-up, and data collection | 3 | This is a retrospective case-control study conducted at The Affiliated Hospital of Qingdao Agricultural University from July 2020 to September 2023. |
| Participants | 6 | (*a*) *Cohort study*—Give the eligibility criteria, and the sources and methods of selection of participants. Describe methods of follow-up  *Case-control study*—Give the eligibility criteria, and the sources and methods of case ascertainment and control selection. Give the rationale for the choice of cases and controls  *Cross-sectional study*—Give the eligibility criteria, and the sources and methods of selection of participants | 3-4 | A total of 39 patients with HSIL and 42 patients with cervical cancer were selected. All patients with cervical cancer were diagnosed by histopathology, excluding other malignant tumors, severe organ diseases and endocrine metabolic diseases, recent use of immunosuppressive drugs and antiviral drugs. The clinical data and follow-up data required by all patients were complete. In addition, 31 women who underwent physical examination in our hospital at the same time were selected as the healthy control group, and the history of cervical cancer, precancerous lesions, chronic cervicitis and other gynecological reproductive system diseases were excluded. |
|  |  | (*b*) *Cohort study*—For matched studies, give matching criteria and number of exposed and unexposed  *Case-control study*—For matched studies, give matching criteria and the number of controls per case | 3 | A total of 39 patients with HSIL and 42 patients with cervical cancer who were treated in our hospital from July 2020 to September 2023 were selected, and 31 women who underwent physical examination in our hospital at the same time were selected as the healthy control group. |
| Variables | 7 | Clearly define all outcomes, exposures, predictors, potential confounders, and effect modifiers. Give diagnostic criteria, if applicable |  | N/A |
| Data sources/ measurement | 8* | For each variable of interest, give sources of data and details of methods of assessment (measurement). Describe comparability of assessment methods if there is more than one group | 3-4 | The clinical data of the patients were collected. ( 1 ) General data : including age, menopause, number of pregnancies, number of deliveries, hypertension, diabetes, etc. ( 2 ) Laboratory indicators : blood routine data including platelet count, absolute neutrophil count, lymphocyte count, and calculate neutrophil / lymphocyte ratio ( NLR ), platelet / lymphocyte ratio ( PLR ), NLR = neutrophil count / lymphocyte count, PLR = platelet count / lymphocyte count ; ( 3 ) Pathological data : The cervical tissue specimens were prepared and stained by the pathologists of our hospital, and the diagnosis was made under the microscope. If necessary, the results of immunohistochemical staining were combined. |
| Bias | 9 | Describe any efforts to address potential sources of bias |  | N/A |
| Study size | 10 | Explain how the study size was arrived at | 3 | This study used a retrospective case-control study. Thirty-nine patients with HSIL and 42 patients with cervical cancer who were treated in our hospital from July 2020 to September 2023 were selected, and 31 women who underwent physical examination in our hospital at the same time were selected as the healthy control group. |

Continued on next page

| Quantitative variables | 11 | Explain how quantitative variables were handled in the analyses. If applicable, describe which groupings were chosen and why | 3 | A total of 39 patients with HSIL and 42 patients with cervical cancer who were treated in our hospital from July 2020 to September 2023 were selected, and 31 women who underwent physical examination in our hospital at the same time were selected as the healthy control group. |
| --- | --- | --- | --- | --- |
| Statistical methods | 12 | (*a*) Describe all statistical methods, including those used to control for confounding | 4-5 | The data obtained from the study were analyzed and processed by SPSS23.0, and the drawing was performed using Gradpad Prism8.0 software. The measurement data were expressed as ( mean ± standard deviation ). The t test was used for comparison between the two groups, and the F test was used for comparison between multiple groups. The count data were expressed as example / %, and the chi-square test was used for comparison. Pearson correlation was used for correlation analysis. P < 0.05 was considered statistically significant. |
|  |  | (*b*) Describe any methods used to examine subgroups and interactions | 5 | The measurement data were expressed as ( mean ± standard deviation ). The t test was used for comparison between the two groups, and the F test was used for comparison between multiple groups. The count data were expressed as example / %, and the chi-square test was used for comparison. Pearson correlation was used for correlation analysis. P < 0.05 was considered statistically significant. |
|  |  | (*c*) Explain how missing data were addressed |  | N/A |
|  |  | (*d*) *Cohort study*—If applicable, explain how loss to follow-up was addressed  *Case-control study*—If applicable, explain how matching of cases and controls was addressed  *Cross-sectional study*—If applicable, describe analytical methods taking account of sampling strategy | 3 | A total of 39 patients with HSIL and 42 patients with cervical cancer who were treated in our hospital from July 2020 to September 2023 were selected, and 31 women who underwent physical examination in our hospital at the same time were selected as the healthy control group. |
|  |  | (*e*) Describe any sensitivity analyses |  |  |
| Results | | | | |
| Participants | 13* | (a) Report numbers of individuals at each stage of study—eg numbers potentially eligible, examined for eligibility, confirmed eligible, included in the study, completing follow-up, and analysed | 5 | Finally, 39 patients with HSIL and 42 patients with cervical cancer were included, and 31 women in the healthy control group were included. |
|  |  | (b) Give reasons for non-participation at each stage |  |  |
|  |  | (c) Consider use of a flow diagram |  |  |
| Descriptive data | 14* | (a) Give characteristics of study participants (eg demographic, clinical, social) and information on exposures and potential confounders | 4 | There was no significant difference in age, menopause, number of pregnancies and other clinical data between cervical cancer, HSIL patients and healthy controls ( P > 0.05 ). |
|  |  | (b) Indicate number of participants with missing data for each variable of interest |  |  |
|  |  | (c) *Cohort study*—Summarise follow-up time (eg, average and total amount) |  |  |
| Outcome data | 15* | *Cohort study*—Report numbers of outcome events or summary measures over time |  |  |
|  |  | *Case-control study—*Report numbers in each exposure category, or summary measures of exposure |  |  |
|  |  | *Cross-sectional study—*Report numbers of outcome events or summary measures |  |  |
| Main results | 16 | (*a*) Give unadjusted estimates and, if applicable, confounder-adjusted estimates and their precision (eg, 95% confidence interval). Make clear which confounders were adjusted for and why they were included | 5 | There was no significant difference in the levels of NLR and PLR in peripheral blood between cervical cancer group and HSIL group ( P > 0.05 ). The levels of PLR and NLR in peripheral blood of cervical cancer group were significantly higher than those of healthy control group ( P < 0.001 ). The levels of PLR and NLR in peripheral blood of cervical cancer group were significantly higher than those of healthy control group ( P < 0.001 ). |
|  |  | (*b*) Report category boundaries when continuous variables were categorized |  | N/A |
|  |  | (*c*) If relevant, consider translating estimates of relative risk into absolute risk for a meaningful time period |  | N/A |

Continued on next page

| Other analyses | 17 | Report other analyses done—eg analyses of subgroups and interactions, and sensitivity analyses | 5-6 | There were statistically significant differences in PLR in peripheral blood of cervical cancer patients with different clinical stages and degree of infiltration of muscle wall ( P < 0.05 ). There were statistically significant differences in peripheral blood NLR among cervical cancer patients with different clinical stages and infiltration degree of muscle wall ( P < 0.05 ). The results of correlation analysis showed that PLR in peripheral blood of patients with cervical cancer was positively correlated with clinical stage and degree of infiltration of muscle wall ( P < 0.05 ), and NLR was positively correlated with clinical stage and degree of infiltration of muscle wall ( P < 0.05 ). |
| --- | --- | --- | --- | --- |
| Discussion | | | | |
| Key results | 18 | Summarise key results with reference to study objectives | 5-6 | Compared with healthy people, the levels of PLR in peripheral blood of patients with HSIL and cervical cancer were further increased, and the levels of NLR and PLR in peripheral blood of patients with cervical cancer were closely related to clinical stage and infiltration of muscle wall. |
| Limitations | 19 | Discuss limitations of the study, taking into account sources of potential bias or imprecision. Discuss both direction and magnitude of any potential bias |  | This study is a single-center retrospective study, and the sample size is small, which may cause some bias to the research results. In the future, the number of cases should be increased, and multiple indicators should be combined to analyze the diagnostic efficacy of cervical cancer and explore its mechanism. |
| Interpretation | 20 | Give a cautious overall interpretation of results considering objectives, limitations, multiplicity of analyses, results from similar studies, and other relevant evidence | 7 | The expression levels of NLR and PLR in peripheral blood of patients with HSIL and cervical cancer are higher, and the levels of NLR and PLR in peripheral blood of patients with cervical cancer are closely related to clinical stage and degree of infiltration of muscle wall, which is helpful for the differential diagnosis of cervical cancer. |
| Generalisability | 21 | Discuss the generalisability (external validity) of the study results | 7 | Our study also observed that the levels of NLR and PLR in peripheral blood of patients with cervical cancer are closely related to the clinical stage and the degree of infiltration of the muscle wall, which is helpful for the differential diagnosis of cervical cancer. |
| Other information | |  | | |
| Funding | 22 | Give the source of funding and the role of the funders for the present study and, if applicable, for the original study on which the present article is based |  | N/A |

*Give information separately for cases and controls in case-control studies and, if applicable, for exposed and unexposed groups in cohort and cross-sectional studies.

**Note:** An Explanation and Elaboration article discusses each checklist item and gives methodological background and published examples of transparent reporting. The STROBE checklist is best used in conjunction with this article (freely available on the Web sites of PLoS Medicine at http://www.plosmedicine.org/, Annals of Internal Medicine at http://www.annals.org/, and Epidemiology at http://www.epidem.com/). Information on the STROBE Initiative is available at www.strobe-statement.org.
